# Crossmodal association of auditory and visual material properties in infants

**DOI:** 10.1038/s41598-018-27153-2

**Published:** 2018-06-18

**Authors:** Yuta Ujiie, Wakayo Yamashita, Waka Fujisaki, So Kanazawa, Masami K. Yamaguchi

**Affiliations:** 1 Research and Development Initiative, Chuo University, 1-13-27, Kasuga, Bunkyo-ku, Tokyo, JP112-8551 Japan; 2 Department of Information Science and Biomedical Engineering, Kagoshima University, 1-21-40 Korimoto, Kagoshima, 890-0065 Japan; 3 Physiological Functions Research Team, Automotive Human Factors Research Center, AIST, 1-1-1, Higashi, Tsukuba, 305-8560 Japan; 4 Department of Psychology, Japan Women’s University, 1-1-1 Nishi-Ikuta, Tama, Kawasaki, Kanagawa 214-8565 Japan; 5 Department of Psychology, Chuo University, 742-1 Higashi-Nakano, Hachioji, Tokyo, 192-0393 Japan

## Abstract

The human perceptual system enables us to extract visual properties of an object’s material from auditory information. In monkeys, the neural basis underlying such multisensory association develops through experience of exposure to a material; material information could be processed in the posterior inferior temporal cortex, progressively from the high-order visual areas. In humans, however, the development of this neural representation remains poorly understood. Here, we demonstrated for the first time the presence of a mapping of the auditory material property with visual material (“Metal” and “Wood”) in the right temporal region in preverbal 4- to 8-month-old infants, using near-infrared spectroscopy (NIRS). Furthermore, we found that infants acquired the audio-visual mapping for a property of the “Metal” material later than for the “Wood” material, since infants form the visual property of “Metal” material after approximately 6 months of age. These findings indicate that multisensory processing of material information induces the activation of brain areas related to sound symbolism. Our findings also indicate that the material’s familiarity might facilitate the development of multisensory processing during the first year of life.

## Introduction

Material properties of objects^1–5^ consist of multisensory information such as color, texture, light field, and hardness. However, multiple modality inputs are not always necessary to judge the category of an object’s material, because one modality input can extract the information of another modality within the human perceptual system. Previous studies demonstrated that, in addition to vision^6,7^, audition provides useful information when humans judge an object’s material by its visual appearance with its impact sound^1^. These results implied the mapping of specific material sounds to the specific visual property of the material. Such mapping has received much attention mostly in the domain of language acquirement, which is known as the sound symbolism^8–11^, which is the multisensory association between shapes and words. For example, the word “kiki” induces the representation of sharper object^7^. From a developmental perspective, the sound symbolism is key to recognizing environmental information, which underlies semantic processing such as language^9–11^. The mapping of specific sound to specific shapes occurs starting from the preverbal stage of infants^10,12,13^. The current study explored (1) whether multisensory processing of material information induces the activation of brain areas related to sound symbolism, and (2) whether the material’s familiarity plays a role in the development of multisensory material perception.

The neural basis of material category perception has been investigated in humans^14,15^ and monkeys^2,3^. Functional magnetic resonance imaging (fMRI) studies revealed cortical activation in the ventral visual pathway in relation to visual-based material perception; the fusiform gyrus in humans^15^ and the inferior temporal cortex in monkeys^2,3^. On the other hand, the activation in the ventro-medial pathway was demonstrated in relation to auditory-based material perception in humans^14^. However, these studies focused on the high-order visual areas, where material information could be processed progressively^2^. Goda *et al*.^2^ revealed that the supramodal neural representation is formed in the posterior inferior temporal cortex in monkeys after simple long-term visuo-haptic experience. The temporal region in the human brain, especially the superior temporal region, is crucial for processing audiovisual information^16–18^. Interestingly, a recent fMRI study demonstrated the activation of the right posterior superior temporal sulcus (STS) during processing the associations of words with shapes and mimetic words with motion, indicating that the right pSTS is a key area for sound symbolism in adults^8^. Even in preverbal infants, the right temporal area is activated when observing a sound symbolic word with a matching shape (e.g., the word “moma” with the round shape^19^). Although the superior temporal region is involved in relatively high-order processing, the mapping of a specific material sound with a visual material property possibly occurs in the STS if material information is processed progressively. Further, the right STS would be sensitive to the mapping of auditory material property with visual material if material category perception is related to sound symbolism^8^.

However, no prior study has directly investigated whether or not the mapping of the auditory material property with visual material occurs in preverbal infants, how it is represented in the infants’ brain, and whether it is lateralized to one hemisphere. The present study sheds light on these questions by using near-infrared spectroscopy (NIRS), a functional brain activity imaging technique, to examine the brain activity in response to audiovisual material matching in 4- to 8-month-old infants. We measured the cerebral hemoglobin concentrations in bilateral temporal brain regions, including the STS area, which is known to be critical for supra-additive processing of auditory and visual information^15^. The results of the present study suggested that the mapping of the auditory material property with visual material in the right temporal region occurs in infants, indicating that audiovisual material information is involved in the relatively high-order processing that envelopes the sound symbolism.

## Results

We measured the brain activity in infants using an NIRS system (ERG-4000; Hitachi Medical). The benefit, variability, and reliability of this method were validated in our previous studies demonstrating the interhemispheric differences in face information processing in infants. In the NIRS experiment, we presented two sets of audiovisual material stimulus. The visual stimulus in one condition set alternated between match and mismatch trials, while the auditory stimulus was constant in both trials. Within each trial, the visual and auditory stimuli were simultaneously presented eight times. Baseline trials consisting of alternating noise images with a white noise preceded a match or mismatch trial. We assigned 32 infants between 4 to 5 months of age and 6 to 8 months of age to either Wood-sound condition or Metal-sound condition. The NIRS responses were subsequently measured in the bilateral temporal regions.

**Figure 1:**
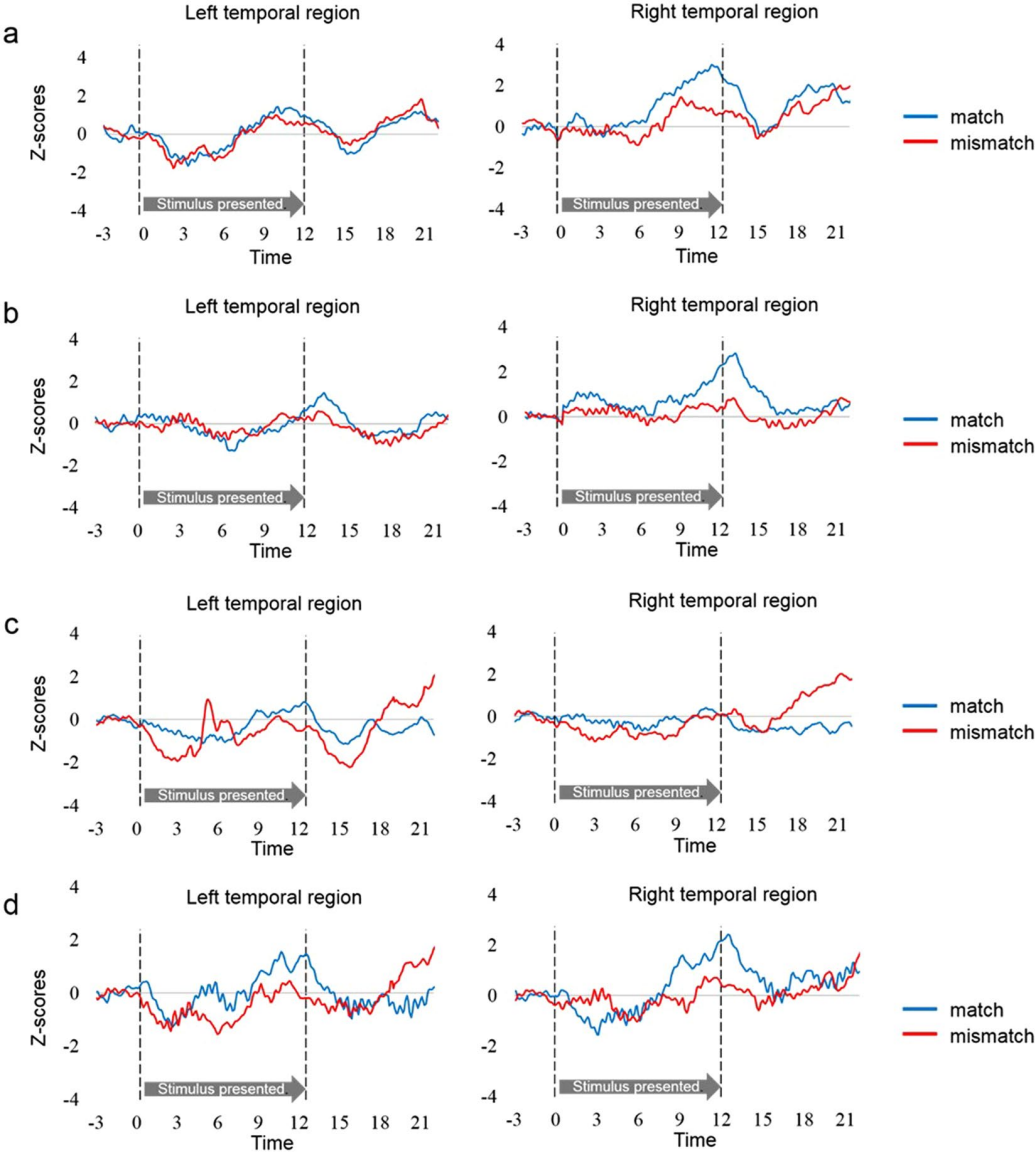
Time course of the changes in the oxygenated hemoglobin (oxy-Hb) concentrations. Oxy-Hb concentrations were averaged in both age groups during each condition in the left and right temporal regions; (**a**) 4- to 5-month-olds for the Wood-sound condition, (**b**) 6- to 8-month-olds for the Wood-sound condition, (**c**) 4- to 5-month-olds for the Metal-sound condition, and (**d**) 6- to 8-month-olds for the Metal-sound condition. Lines in blue and lines in red represent the mean Z-score during the match and mismatch trials, respectively. The vertical dashed lines at 0 and 12.4 s indicate the onset and offset of the test stimulus presentation, respectively.

Hemodynamic data were obtained from 16 infants in total (eight 4- to 5-month-olds and eight 6- to 8-month-olds) in two sound conditions. Each sound condition consisted of more than three valid trials in the match and mismatch trials. We normalized the raw data of the hemodynamic responses using the mean and the standard deviation (SD) of the baseline period (details are in Methods) for each channel and each participant before applying statistical analyses, because the raw data could not be averaged directly across participants and channels. Subsequently, we averaged the Z-scores of oxygenated hemoglobin (oxy-Hb) across 12 channels in each of the left and right hemispheres, and compared them against the baseline. Figure 1 illustrates the time course of the average changes of oxy-Hb during the presentation of match and mismatch trials, respectively for each condition and age group (results of deoxygenated Hb [deoxy-Hb] and total-Hb changes are provided in the Supplementary Fig. S1 and Fig. S2). In the Wood-sound condition, increased concentrations of oxy-Hb in the right temporal regions during the match trials, but not during the mismatch trials, were observed in both age groups. In the Metal-sound condition, similar results were observed only in the group of 6- to 8-month-olds. This increased activation in the match trials occurred at around 9 s after the stimulus onset, reached the peak subsequently, and started to return toward the baseline level at around the end of the test period.

**Figure 2:**
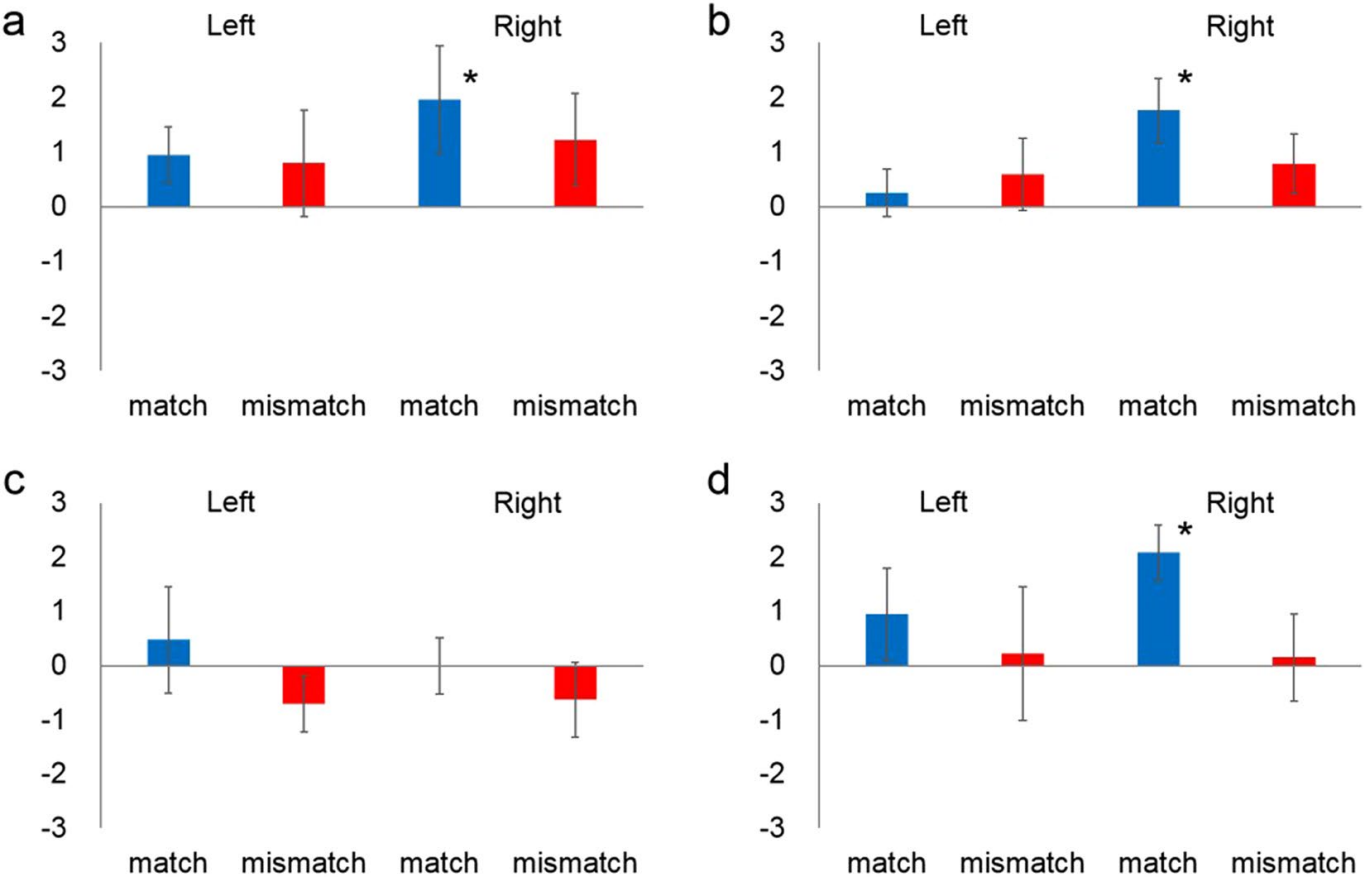
Mean Z-scores of near-infrared spectroscopy response in both age groups for each of the left temporal (Left) and right temporal (Right) regions. (**a**) 4- to 5-month-olds for the Wood-sound condition, (**b**) 6- to 8-month-olds for the Wood-sound condition, (**c**) 4- to 5-month-olds for the Metal-sound condition, and (**d**) 6- to 8-month-olds for the Metal-sound condition. Each bar represents the mean Z-score of oxygenated hemoglobin (oxy-Hb) averaged across 9–13 s in the stimulus onset latency. Bars in blue and bars in red represent the results for the match and the mismatch conditions, respectively. The error bars represent ± 1 standard error of the mean. Asterisks indicate the significance level of the statistical differences: *P < 0.05 and **P < 0.01.

To test the asymmetric activation in the right hemisphere for processing of audiovisual material matching, we performed statistical analysis on the mean Z-scores during the 9–13-s period after stimulus onset in the left and right temporal regions (Fig. 2). We then conducted a three-factor repeated-measure analysis of variance (ANOVA) on the oxy-Hb data by taking groups (4- to 5-month-olds *vs* 6- to 8-month-olds) as a between-participant factor, and congruency (match *vs*. mismatch) as well as measurement region (left *vs*. right) as within-participant factors. In the Wood-sound condition, the ANOVA revealed a significant main effect of the measurement region (*F*[1,14] = 5.17, *p* = 0.039, η2 = 0.04) and a marginally significant interaction between measurement region and congruency (*F*[1,14] = 4.21, *p* = 0.059, η2 = 0.02). A simple main effect analysis revealed a significant activation of oxy-Hb during match trials in the right hemisphere rather than in the left hemisphere (*F*[1,14] = 7.64, *p* = 0.015, η2 = 0.14). No significant main effects or interactions were observed for the Metal-sound condition.

Next, we conducted a two-tailed one sample *t*-test against zero response (baseline) in order to see whether each temporal region was activated in response to audiovisual material matching. In the Wood-sound condition, the concentration of oxy-Hb in the right temporal region increased significantly during the match trials in both groups (4- to 5-month-olds: *M* = 1.96, *SD* = 2.77, standard error [*SE*] = 0.91, *t* [7] = 2.37, *p* = 0.049, *d* = 0.71; 6- to 8-month-olds: *M* = 1.76, *SD* = 1.66, *SE* = 0.59, *t* [7] = 2.91, *p* = 0.02, *d* = 1.06), but not during the mismatch trials (4- to 5-month-olds: *M* = 1.22, *SD* = 2.37, *SE* = 0.84, *t* [7] = 1.06, *p* = 0.32, *d* = 0.51; 6- to 8-month-olds: *M* = 0.78, *SD* = 1.55, *SE* = 0.55, *t* [7] = 0.78, *p* = 0.46,, *d* = 0.50). In contrast, in the left temporal region, no significant increases in the concentration of oxy-Hb were observed either in the match trials (4- to 5-month-olds: *M* = 0.94, *SD* = 1.45, *SE* = 0.51, *t* [7] = 1.94, *p* = 0.09, *d* = 0.65; 6- to 8-month-olds: *M* = 0.26, *SD* = 1.23, *SE* = 0.44, *t* [7] = 0.65, *p* = 0.54, *d* = 0.21) or in the mismatch trials (4- to 5-month-olds: *M* = 0.79, *SD* = 2.75, *SE* = 0.97, *t* [7] = 0.58, *p* = 0.58, *d* = 0.29; 6- to 8-month-olds: *M* = 0.59, *SD* = 1.88, *SE* = 0.67, *t* [7] = 0.78, *p* = 0.46, *d* = 0.31). In the Metal-sound condition, the concentration of oxy-Hb in the right temporal region was increased during the match trials in the group of 6- to 8-month-olds (*M* = 2.08, *SD* = 1.44, *SE* = 0.51, *t* [7] = 2.66, *p* = 0.03, *d* = 1.44), but not in the 4- to 5-month-olds (*M* = −0.01, *SD* = 1.45, *SE* = 0.51, *t* [7] = 0.14, *p* = 0.89, *d* = 0.01). No increases were observed during the mismatch trials in both age groups (4- to 5-month-olds: *M* = −0.63, *SD* = 1.95, *SE* = 0.69, *t* [7] = 0.11, *p* = 0.92, *d* = 0.32; 6- to 8-month-olds: *M* = 0.15, *SD* = 2.27, *SE* = 0.80, *t* [7] = 0.35, *p* = 0.73, *d* = 0.07). In the left temporal region, the concentration of oxy-Hb did not increase significantly during the match trials (4- to 5-month-olds: *M* = 0.47, *SD* = 2.79, *SE* = 0.99, *t* [7] = 0.49, *p* = 0.64, *d* = 0.17; 6- to 8-month-olds: *M* = 0.95, *SD* = 2.40, *SE* = 0.85, *t* [7] = 1.37, *p* = 0.21, *d* = 0.40) nor during the mismatch trials (4- to 5-month-olds: *M* = −0.71, *SD* = 1.45, *SE* = 0.51, *t* [7] = 0.40, *p* = 0.70, *d* = 0.49; 6- to 8-month-olds: *M* = 0.22, *SD* = 3.49, *SE* = 1.23, *t* [7] = 0.29, *p* = 0.78, *d* = 0.06).

**Figure 3:**
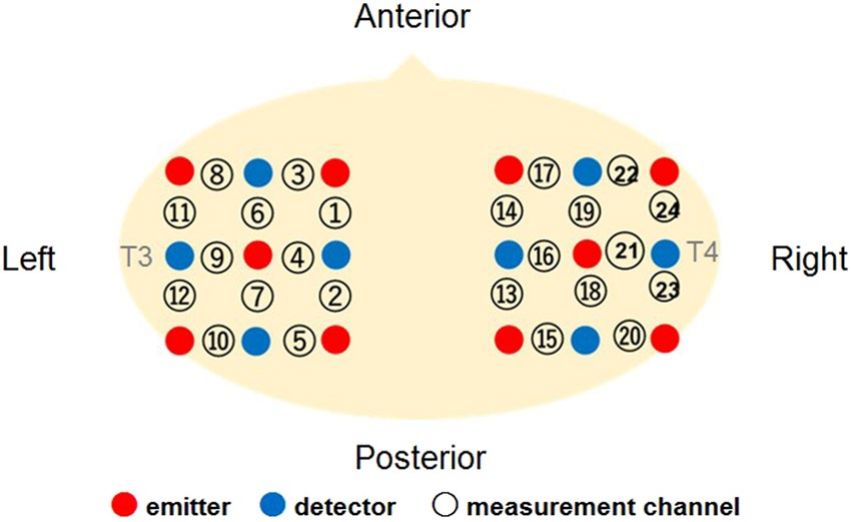
Location of the measurement channels in the current study. The probe holders were placed on the right and left temporal regions, which were placed at the T3 and T4 position of the international 10–20 system. These probe positions were included in the bilateral superior temporal sulcus regions. The distance between the emitter and detector probes was set at 2 cm.

A further analysis was conducted to examine the cortical area that potentially exhibited brain activity related to audiovisual material matching. Based on the locations of 10–20 cortical projection points, individual channels in fNIRS measurement can be estimated in the anatomical brain area^21^, even in infants’ brain^22^. In our setting of channels’ location (Fig. 3), the right superior temporal channels (ch 10 and ch 20) can be considered to reflect the activation of the right superior temporal sulcus, according to the anatomical regions for the projection of each fNIRS channel in Loyd-Fox *et al*. (2014)^22^. We then conducted one sample t-test against the zero response (baseline) on the Z-scores of oxy-Hb for each channel separately. In the Metal-sound condition, the right superior temporal channel (ch 20) increased significantly during the match trials (*t* [7] = 3.16, *p* = 0.02, *d* = 1.12) rather than the mismatch trials (*t* [7] = 1.22, *p* = 0.26, *d* = 0.43). Similar results were found in the Wood-sound condition, although at a marginally significant level (match trials: *t* [7] = 2.32, *p* = 0.054, *d* = 0.82; mismatch trials: *t* [7] = 0.31, *p* = 0.77, *d* = 0.11). The responses from this channel (ch 20) could be assumed to be associated with the activation of the right superior temporal sulcus^21,22^, which is related to the processing of sound symbolism^8^. To summarize, the individual channel analysis indicated significant differences in oxy-Hb responses in the temporal regions between trials, which suggests that audiovisual material matching is represented in the right superior temporal area in infants already at the age of 6 to 8 months.

To our knowledge, the present study demonstrated for the first time that the processing of audiovisual material matching is already developed in the preverbal infants. Regardless of the material type (wood or metal), the presentation of audiovisual match stimuli increased the concentrations of oxy-Hb in the right hemisphere. The measurement region in this study included the right posterior STS, which is sensitive to the processing of sound symbolism^8^. Our findings indicated that (1) the association between the visual material property and the auditory material property of an object is already developed in infants at 4- to 5-month of age, and that (2) the brain activity in the right hemisphere related to sound symbolism is modulated depending on the congruency of auditory and visual material properties of the object.

The development of the association of multisensory material properties possibly depends on the material’s familiarity, which is reflected by the fact that younger infants are less familiarized by the material properties of metal. Goda *et al*.^2^ demonstrated that the visuo-haptic experience of exposing materials formed the specific neural representation to materials in the posterior inferior temporal cortex in monkeys. In addition, infants are unable to discriminate the surface glossiness of objects until around 7 months of age^4,5^. Furthermore, 7- to 8-month-old infants can discriminate between glossy and matte objects, probably based on the surface representation as glossiness rather than the property of image statistics such as contrast or skewness^4^. In our results, 4- to 5- month-old infants showed activation in the right hemisphere in the Wood-sound condition rather than in the Metal-sound condition. Based on previous studies^2,4^ and on our present results, infants acquire the sound-object association for a property of the “Metal” material later than for the “Wood” material, since infants form the visual property of “Metal” material after approximately 6 months of age. In other words, 6- to 8-month-old infants can discriminate the congruency of audiovisual material property in both the Wood-sound and the Metal-sound conditions. To confirm this, we further conducted the control experiment using the familiarization paradigm (details are provided in the supplementary section). The results suggested that 6- to 8-month-old infants discriminated novel (the audiovisual mismatch stimuli) from familiarized stimuli (the audiovisual match stimuli) in both the Wood-sound and the Metal-sound conditions. Although the sample used for fNIRS and behavioral experiments was different, our behavioral data strongly supported our assumption that the processing of audiovisual material matching is already developed in 6- to 8-month-old infants, regardless of material familiarity.

Previous studies revealed that the activation in the right STS was sensitive to a semantic network for processing the sound-object association^8^, which is known as sound symbolism^9–13^. Such asymmetric activation related to sound symbolism was predicted even in preverbal infants^19^. In line with this, we found that the asymmetric activation in the right hemisphere reflected multisensory processing for material properties of objects. There is ample evidence that the posterior superior temporal region is sensitive to the processing of audiovisual inputs rather than the processing of auditory or visual input separately^16–18^. An fMRI study in adults revealed the functional dissociation between the left and the right hemispheres that are involved in the semantic processing of words and environmental sounds. In particular, the environmental sounds induced the activation of the right posterior STS, while words activated the left posterior STS^23^. Interestingly, such activation was observed when participants made semantic decision (i.e., categorization) but not when they listened to these sounds passively. Furthermore, the sound symbolism, which operates semantic processing rather than low-level audiovisual binding, induced the activation of the right STS in adults^8^ and of the right temporal hemisphere in infants^19^. Indeed, the infants’ semantic processing of audiovisual inputs such as speech is reported to be formed across the half of the first year^24–26^. Our results supported the idea that infants, in preverbal stages, have the conceptual association between the specific sound and visual information, which leads to and facilitates language acquisition^9^.

In summary, the current study, for the first time, provided evidence that infants exhibited the mapping of specific material sounds to specific property of visual material similarly to adults^1^ prior to the formation of the sound symbolism. Furthermore, our results indicate that such development might depend on a material’s familiarity.

## Methods

### Participants

The participants were 32 healthy infants aged 4–8 months (19 girls and 13 boys, mean age = 168 days, age range 114–232 days). Participants were divided into two age groups; 4- to 5-month-olds (11 girls and 5 boys, mean age = 137 days, age range 114–163 days) and 6- to 8- month-olds (8 girls and 8 boys, mean age = 200 days, age range 169–232 days). An additional seven infants were tested, but were excluded from the analysis because of fussiness (n = 2), motion artifacts (n = 2), or insufficient number of available trials (n = 3; at least three trials for each experimental condition). This experiment was conducted according to the Declaration of Helsinki and was approved by the Ethical Committee of Chuo University. Parents gave prior written informed consent for their children’s participation and for publication in an online open-access.

### Apparatus

A 21-inch color cathode ray tube (CRT) display with a resolution of 1,024 × 768 pixels was used to present the visual stimuli. The display was placed in front of the infant at a distance of 40 cm. A pinhole camera was set below the display to monitor the infant’s looking behavior. The audio stimuli were presented at a sound pressure level of approximately 60 dB through two loudspeakers located on the left and right sides of the display.

The Hitachi ETG-4000 system (Hitachi Medical, Chiba, Japan) was used to record the hemodynamic response simultaneously from 24 channels, with 12 channels for each the right and left temporal area. The instrument generated two different wavelengths (695 and 830 nm) and measured the time course of changes in oxy-Hb, deoxy-Hb, and total-Hb with a 0.1-s time resolution. We used a pair of probes, each containing nine optical fibers (3 × 3 arrays) with five light emitters and four detectors. The optical fibers of each probe were kept in place with a soft silicon holder and the inter-fiber distance was set at 2 cm. According to the International 10–20 EEG system, the center of each probe was placed at the T3 and T4 position for the measurement of the bilateral temporal regions^18^. After positioning the probes, the experimenter checked whether the signals of the channels were appropriate to measure the hemodynamic responses via the ETG-4000 system, which automatically detects whether or not the probes were contacting the infant’s scalp correctly. The channels were rejected from the analysis if adequate contact between the fibers and scalp could not be achieved because of interference from hair.

### Stimuli and procedure

We used the audiovisual material stimuli of two material categories, namely wood and metal, which were developed by Fujisaki *et al*.^1^. The visual stimuli were computer-generated movies (800 × 600 pixels) where a human right hand with a small stick claps a wood or metal object. The auditory stimuli were the impact sounds of the real objects of each material. The visual and auditory stimuli were combined to create two match and two mismatch stimuli. The congruency of the stimuli was based on the material sound. For example, the wood-sound match stimulus was the impact sound of wood with a movie of clapping the wood object, while the wood-sound mismatch stimulus was the wood sound with a movie of clapping the metal object. The sequence of the stimuli presentation consisted of two test periods and a baseline period. As stimuli of the test periods, the match and mismatch stimuli were used. The duration of each stimulus was 1.55 s. The match and mismatch stimuli were presented eight times each, in alternating trials. The duration of the test periods was 12.4 s. During the inter-trial interval (a baseline period), dynamic random dot patterns (800 × 600 pixels) with an auditory white noise were displayed repetitively once per 1.6 s. The inter-trial interval was controlled by the experimenter, and its duration was at least 12.8 s.

Each infant was seated on her (or his) parent’s or an experimenter’s lap. The viewing distance was approximately 40 cm. The infants looked at the stimuli passively while their brain activity was recorded. They were allowed to look at the stimuli as long as they were willing to. Their behavior was recorded digitally throughout the experiment.

### Data analysis

According to the exclusion criteria of previous studies^27,28^, we removed trials from analysis if (1) the infants’ looking time in the test period was less than 6 s or if they became fussy, (2) the infant looked back to the experimenter’s or parent’s face during the preceding baseline period, or (3) motion artifacts were detected by the analysis of sharp changes in the time courses of the raw oxy-Hb data.

The raw Hb data changes from the individual channels were digitally band-pass-filtered at 0.02–1.0 Hz to remove longitudinal signal drift and noise from the instrument. We averaged the raw data of each channel across trials within a participant in a time series from 3 s before the test trial onset to 10 s after the test trial offset. From the time series of raw data of oxy-, deoxy-, and total-Hb, we calculated the Z-scores at each time point, separately for the matching and mismatching conditions. The Z-scores, as the difference of the means between the baseline and test condition, were calculated using the following formula:

$$d=(Test-{M}_{baseline})/S$$

where *Test* represents the raw data values at each time point during test periods, *M baseline* represents the mean of raw data during the baseline period of 3 s immediately before the beginning of each test period, and *S* indicates the standard deviation of the raw data during the same range of baseline periods as *M baseline*.

### Ethics

This experiment was conducted according to the Declaration of Helsinki and was approved by the Ethical Committee of Chuo University. Parents gave prior written informed consent for their children’s participation and for publication in an online open-access.

## Electronic supplementary material

Supplementary Information
