# Supplementary material for: Crossmodal association of auditory and visual material properties in infants
Source: Sci Rep. 2018 Jun 18;8:9301. doi: 10.1038/s41598-018-27153-2 (PMC6006328; doi:10.1038/s41598-018-27153-2)
Supplement: Supplementary file 1 — Supplementary Information [file 41598_2018_27153_MOESM1_ESM.docx]

Title

Crossmodal association of auditory and visual material properties in infants.

Author lists

Yuta Ujiie^1^, Wakayo Yamashita^2^, Waka Fujisaki^3^, So Kanazawa^4^, and Masami K Yamaguchi^5^

Author affiliations

^1^ Research and Development Initiative, Chuo University, 1-13-27, Kasuga, Bunkyo-ku, Tokyo, JP112-8551, Japan.

^2^ Department of Information Science and Biomedical Engineering, Kagoshima University, 1-21-40 Korimoto, Kagoshima, 890-0065, Japan.

^3^ Sensory and Perceptual Information Design Research Group, Human Information Research Institute, AIST, 1-1-1, Umezono, Tsukuba, 305-8560, Japan.

^4^ Department of Psychology, Japan Women’s University, 1-1-1 Nishi-Ikuta, Tama, Kawasaki, Kanagawa 214-8565, Japan.

^5^ Department of Psychology, Chuo University, 742-1 Higashi-Nakano, Hachioji, Tokyo 192-0393, Japan.

The corresponding author

Yuta Ujiie, Research and Development Initiative, Chuo University, 1-13-27, Kasuga, Bunkyo-ku, Tokyo, JP112-8551, Japan.

E-mail: yuta.ujiie.160330@gmail.com

Supplemental Figures


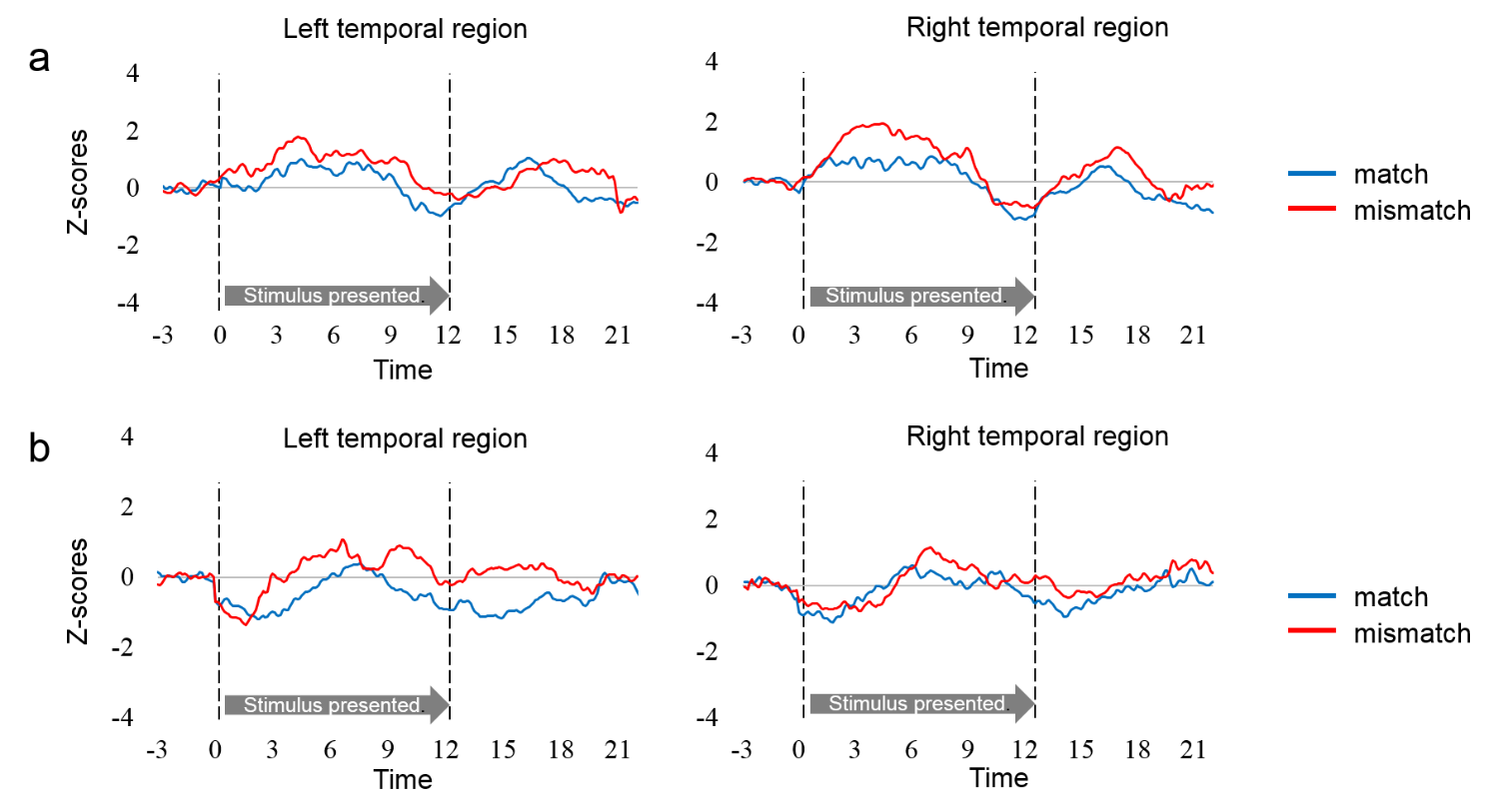


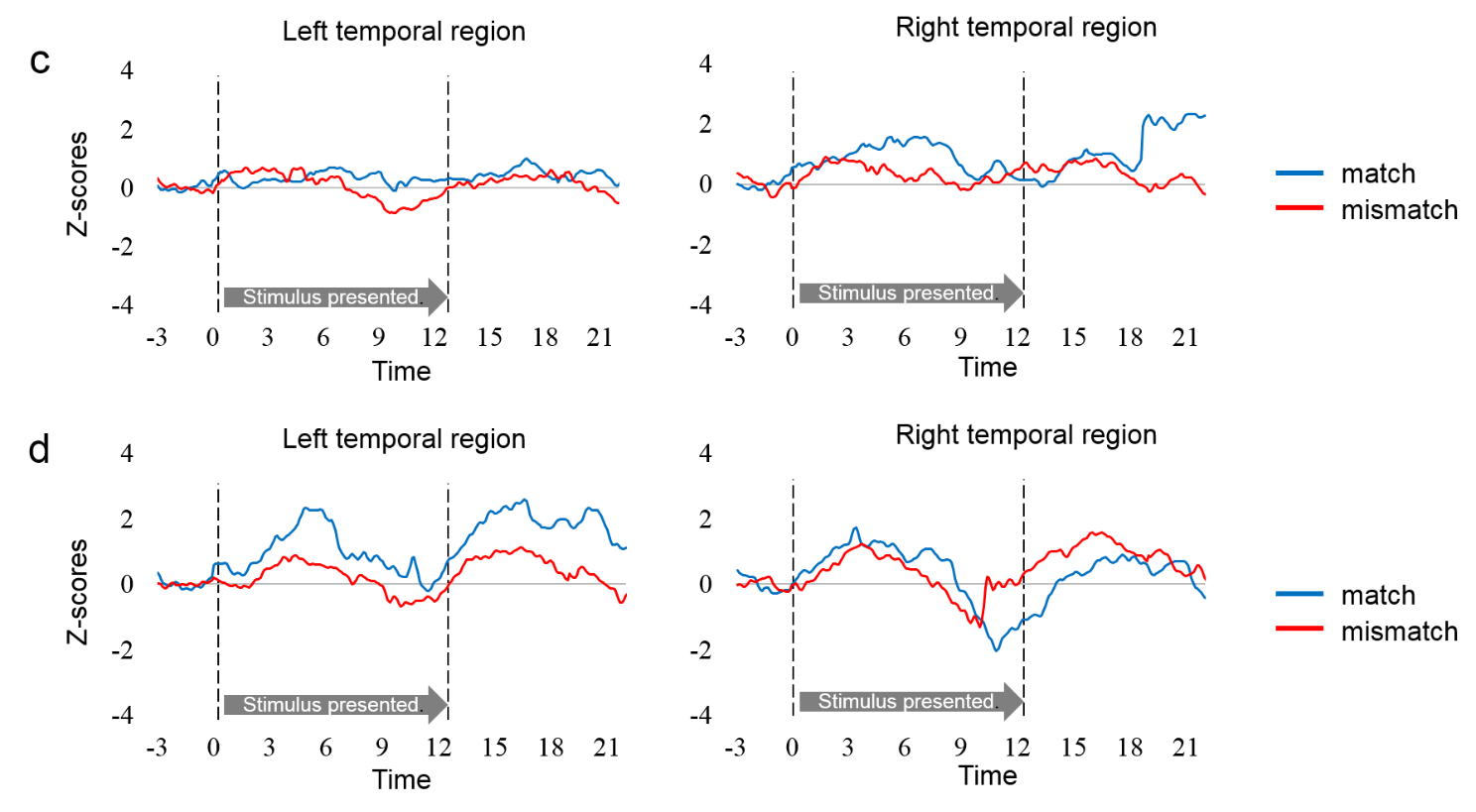


Figure S1. Time course of the changes in the deoxygenated hemoglobin (deoxy-Hb) concentrations. Deoxy-Hb concentrations were averaged in both age groups during each condition in the left and right temporal regions; (a) 4- to 5-month-olds for the Wood-sound condition, (b) 6- to 8-month-olds for the Wood-sound condition, (c) 4- to 5-month-olds for the Metal-sound condition, and (d) 6- to 8-month-olds for the Metal-sound condition. Lines in blue and lines in red represent the mean Z-score during the match and mismatch trials, respectively. The vertical dashed lines at 0 and 12.4 s indicate the onset and offset of the test stimulus presentation, respectively.


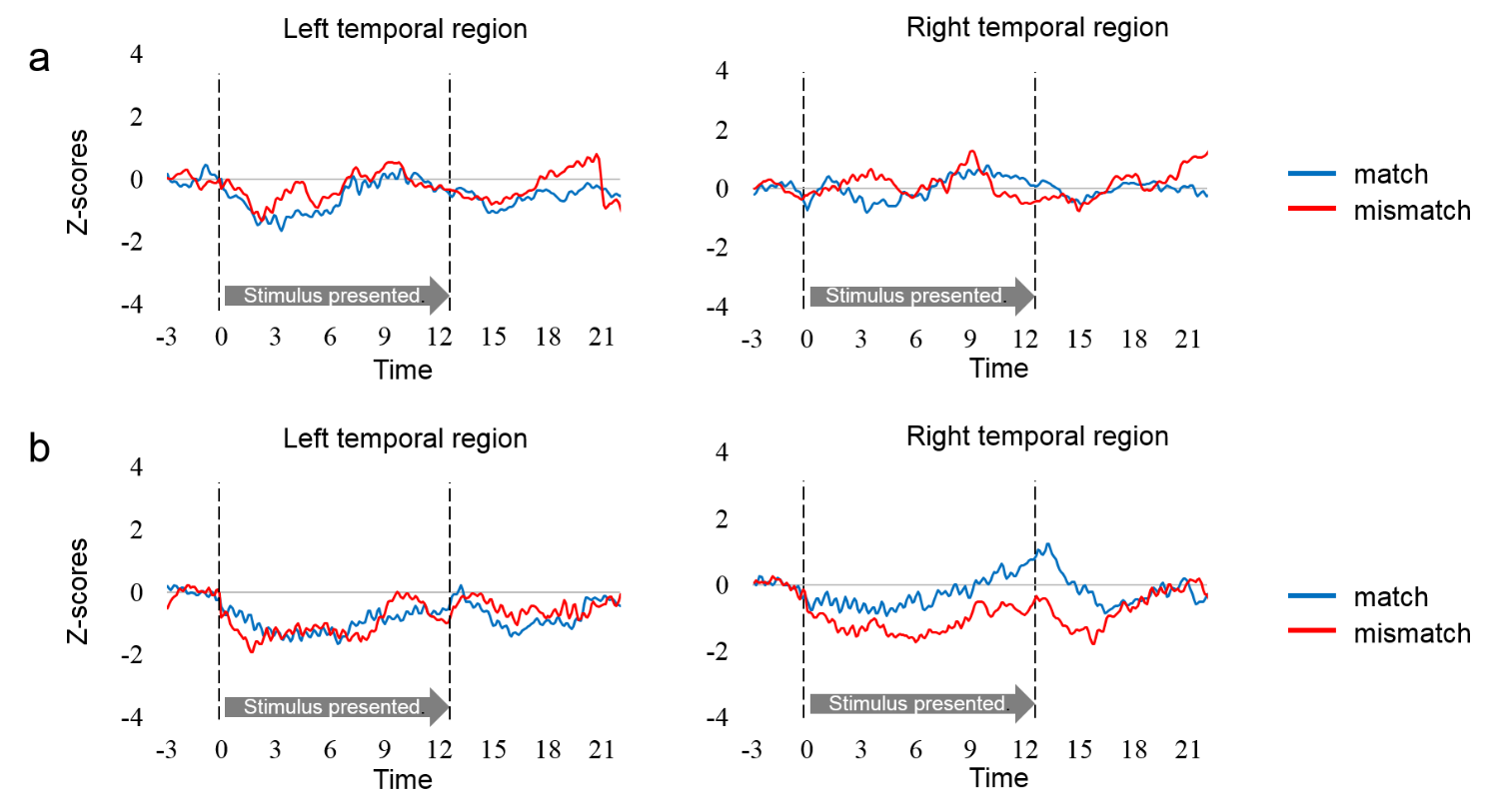


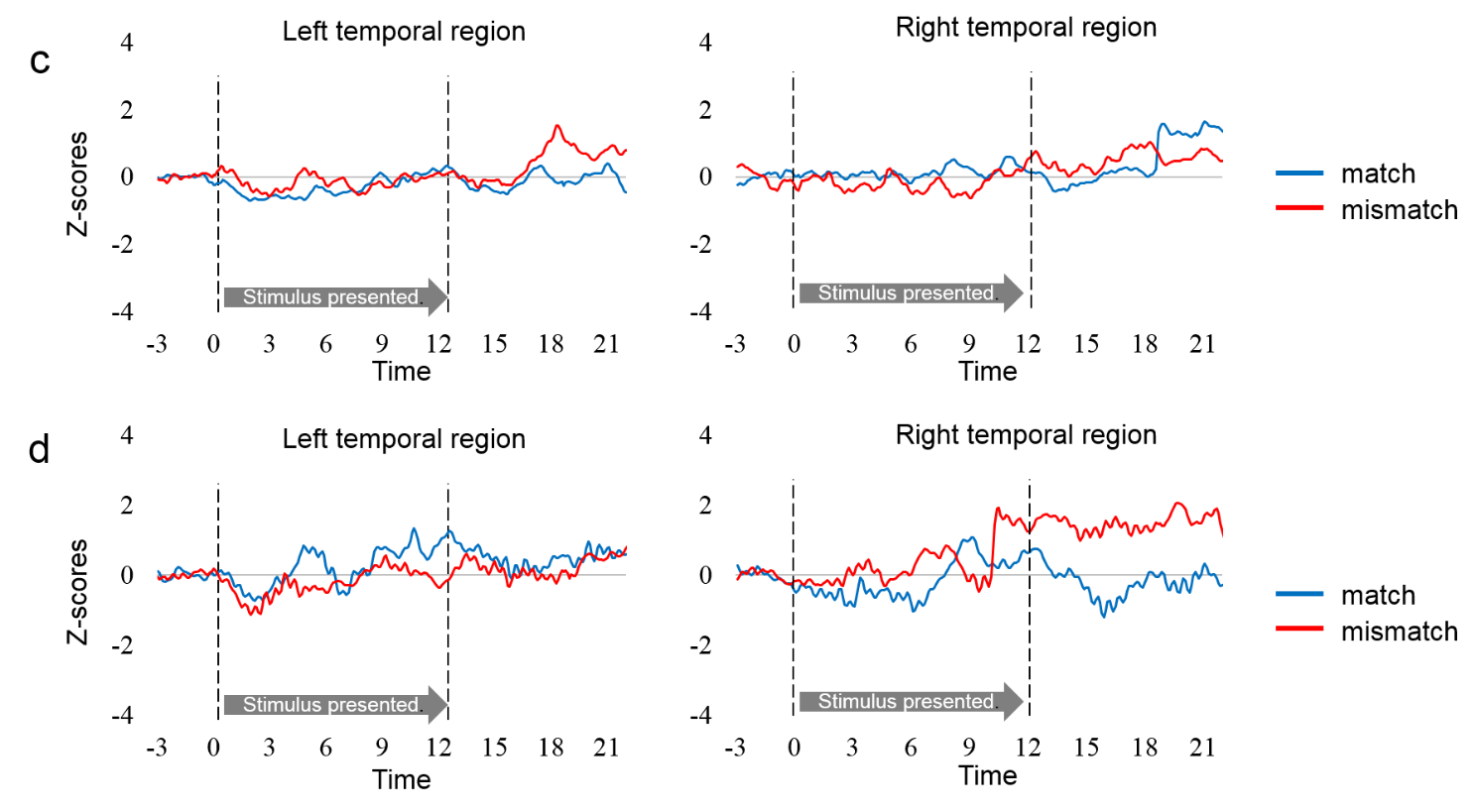


Figure S2. Time course of the changes in the total-Hb concentrations. Total-Hb concentrations were averaged in both age groups during each condition in the left and right temporal regions; (a) 4- to 5-month-olds for the Wood-sound condition, (b) 6- to 8-month-olds for the Wood-sound condition, (c) 4- to 5-month-olds for the Metal-sound condition, and (d) 6- to 8-month-olds for the Metal-sound condition. Lines in blue and lines in red represent the mean Z-score during the match and mismatch trials, respectively. The vertical dashed lines at 0 and 12.4 s indicate the onset and offset of the test stimulus presentation, respectively.


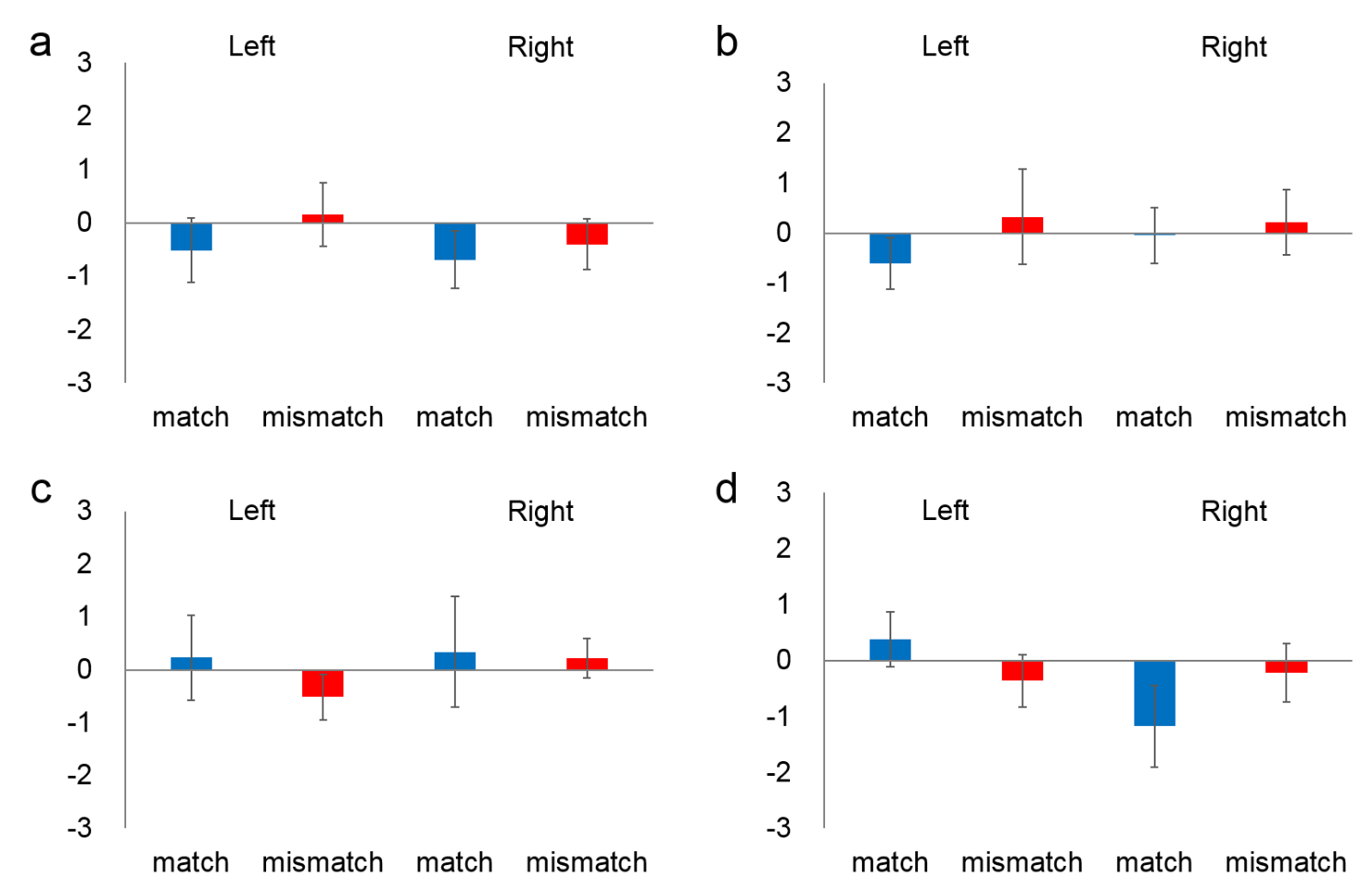
Figure S3. Mean Z-scores of near-infrared spectroscopy (NIRS) response in both age groups for each of the left temporal (Left) and right temporal (Right) regions. (a) 4- to 5-month-olds for the Wood-sound condition, (b) 6- to 8-month-olds for the Wood-sound condition, (c) 4- to 5-month-olds for the Metal-sound condition, and (d) 6- to 8-month-olds for the Metal-sound condition. Each bar represents the mean Z-score of deoxygenated hemoglobin (deoxy-Hb) averaged across 9–13 s in the stimulus onset latency. Bars in blue and bars in red represent the results for the match and the mismatch conditions, respectively. The error bars represent ±1 standard error of the mean. No significant increases of deoxy-Hb were observed during the match and mismatch trials in all conditions of both age groups.


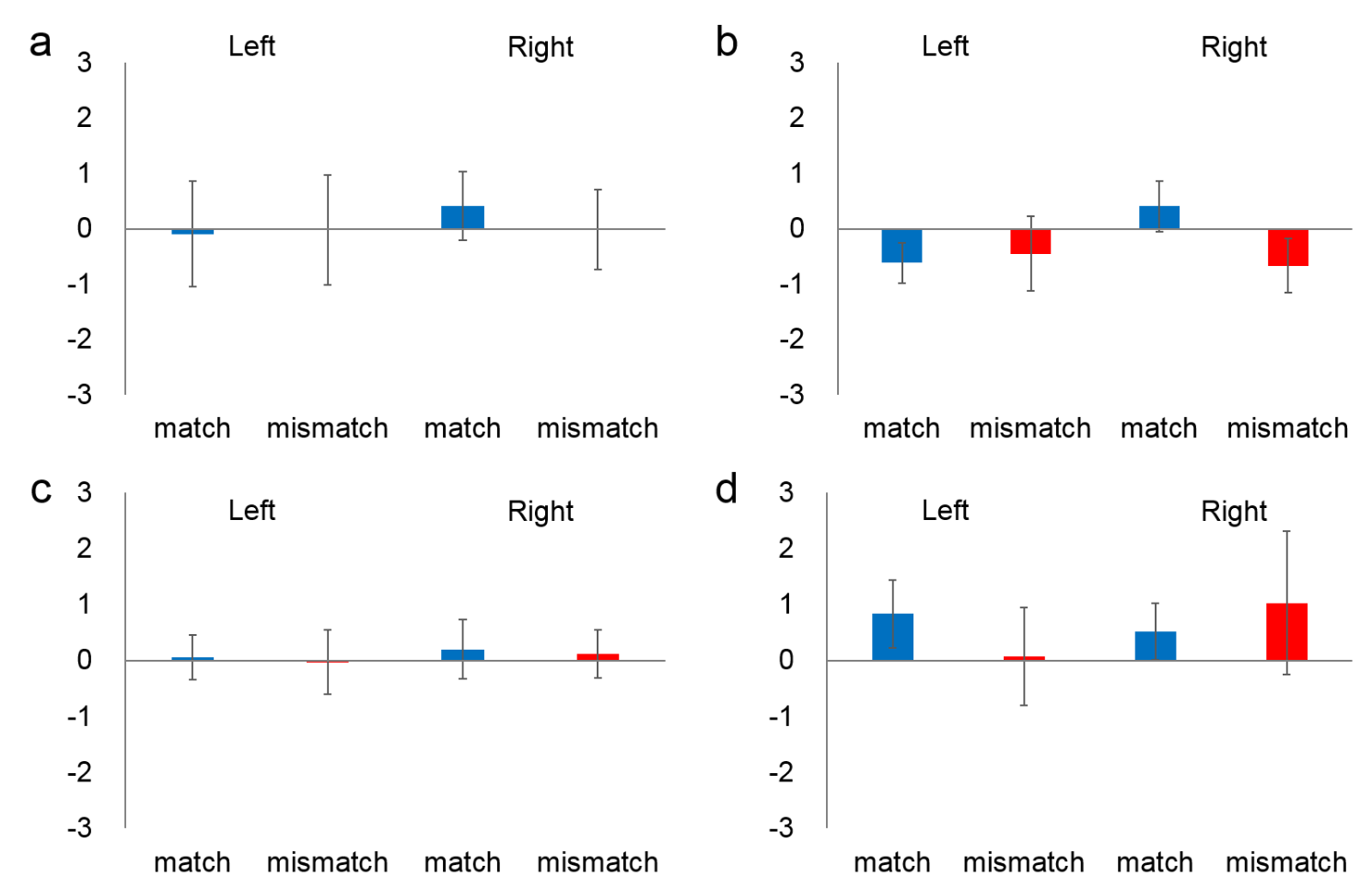
Figure S4. Mean Z-scores of NIRS response in both age groups for each of the left temporal (Left) and right temporal (Right) regions. (a) 4- to 5-month-olds for the Wood-sound condition, (b) 6- to 8-month-olds for the Wood-sound condition, (c) 4- to 5-month-olds for the Metal-sound condition, and (d) 6- to 8-month-olds for the Metal-sound condition. Each bar represents the mean Z-score of deoxygenated hemoglobin (deoxy-Hb) averaged across 9–13 s in the stimulus onset latency. Bars in blue and bars in red represent the results for the match and the mismatch conditions, respectively. The error bars represent ±1 standard error of the mean. No significant increases of total-Hb were observed during the match and mismatch trials in all conditions of both age groups.

Supplementary experiment

To confirm that the processing of audiovisual material matching is developed in 6- to 8-month-olds, we performed the following experiment using the familiarization paradigm.

Methods

Participants

The participants were 18 healthy infants aged 6–8 months (mean age = 208.6 days, age range 182–242 days). This experiment was conducted according to the Declaration of Helsinki and was approved by the Ethical Committee of Chuo University. Parents gave prior written informed consent for their children’s participation.

Apparatus

The visual stimuli were displayed on a 21-inch CRT monitor with a resolution of 1,024 × 768 pixels. The monitor was placed in front of the infant at a distance of 40 cm. The audio stimuli were presented at a sound pressure level of approximately 60 dB through two loudspeakers located on the left and right sides of the display. To record the infant’s looking behavior, a pinhole camera was set below the display.

Stimuli and procedure

We used the same stimulus sets of audiovisual material matching used in the fNIRS experiment. The congruency of the stimuli was based on the material sound. The experiment task consisted of a familiarization phase and test phase. In the familiarization phase, infants were familiarized to the sequence repeated the presentation of an audiovisual match stimulus. In the test phase, the sequence of the familiarized stimulus (the novel stimulus) was presented, which was followed by the sequence of the novel stimulus (the familiarization stimulus), in order to test for discriminating the congruency of the stimuli. We applied a between-participants design, and assigned each 8 infants to either the Wood-sound condition or Metal-sound condition.

Familiarization phase.

The familiarization phase consisted of 6 trials which lasted 20 seconds. The presentation of an animated audiovisual attention getter in the center of the screen was preceded by each trial. In order to ensure that all infants begin to familiarize with the stimuli from the same location, the next trial was progressed only when the infant looked at the fixation.

Test phase.

The test phase consisted of two trials; the familiarized and novel trial. The familiarized trial was the sequence of audiovisual match stimuli, while the novel trial was the sequence of audiovisual mismatch stimuli. Each trial lasted 20 seconds which was preceded by the presentation of the fixation cue in the center of monitor. The order of the presentation of two trials was randomly counterbalanced across the infants.

Each infant was seated on her (or his) parent’s lap. The viewing distance was approximately 40 cm. The infants looked at the stimuli on the monitor without any active task. Their behavior was recorded digitally throughout the experiment.

Results and discussion

Familiarization trials.

The mean total looking time across the first half and second half of familiarization trials in the Wood-sound condition and Metal-sound condition were summarized in Table S1. A mixed ANOVA with trials as a within-participants factor and the stimulus conditions as a between-participants factor showed a significant main effect of trials (*F*[1,16] = 28.69, *p* < .01, η2 = .39). There were no significant main effect and interaction. These results indicate that all infants familiarized with the audiovisual match stimuli without any differences in decreased looking times during the familiarization phase between the metal and wood conditions.

Test trials.

In the Metal-sound condition, the mean total looking times during the novel trial was 13.63 s (*SD*=2.41, *SE*=.57), while that during the familiarized trial was 10.51 s (*SD*=2.80, *SE*=.66). In the Wood-sound condition, the mean total looking times during the novel trial was 14.99 s (*SD*=3.69, *SE*=.87), while that during the familiarized trial was 12.52 s (*SD*=4.20, *SE*=.99). To test whether infants discriminate the congruency of two audiovisual stimuli, we conducted two-tailed one-sample t test. This analysis revealed that infants showed a significant preference for the novel stimuli (audiovisual mismatch stimuli) in both wood and metal conditions ( *t* [8] = 2.94, *p* = .02, *t* [8] = 3.73, *p* = .006) . Further, a mixed ANOVA, with trials as a within-participants factor and the stimulus conditions as a between-participants factor, showed no difference in total looking time across the test phase between the Metal-sound condition and the Wood-sound condition (*F*[1,16] = 1.31, *n.s.*). These results suggest that 6- to 8-month-old infants discriminated the novel (the audiovisual mismatch stimuli) from familiarized stimuli (the audiovisual match stimuli). These behavioral data support our findings of fNIRS experiment, indicating that the processing of audiovisual material matching is already developed in the preverbal infants before the formation of sound symbolism^S1, S2^.

Table S1. Mean total looking times (sec) across the first half and second half of familiarization trials in both the Wood-sound condition and the Metal-sound condition.

|  |  | Familiarization phase | |
| --- | --- | --- | --- |
|  |  | the first half of trials | the second half of trials |
| Wood-sound condition | | |  |
|  | Mean | 17.3 | 13.2 |
|  | SD | 1.76 | 3.92 |
| Metal-sound condition | | |  |
|  | Mean | 16.9 | 12.6 |
|  | SD | 1.99 | 3.14 |


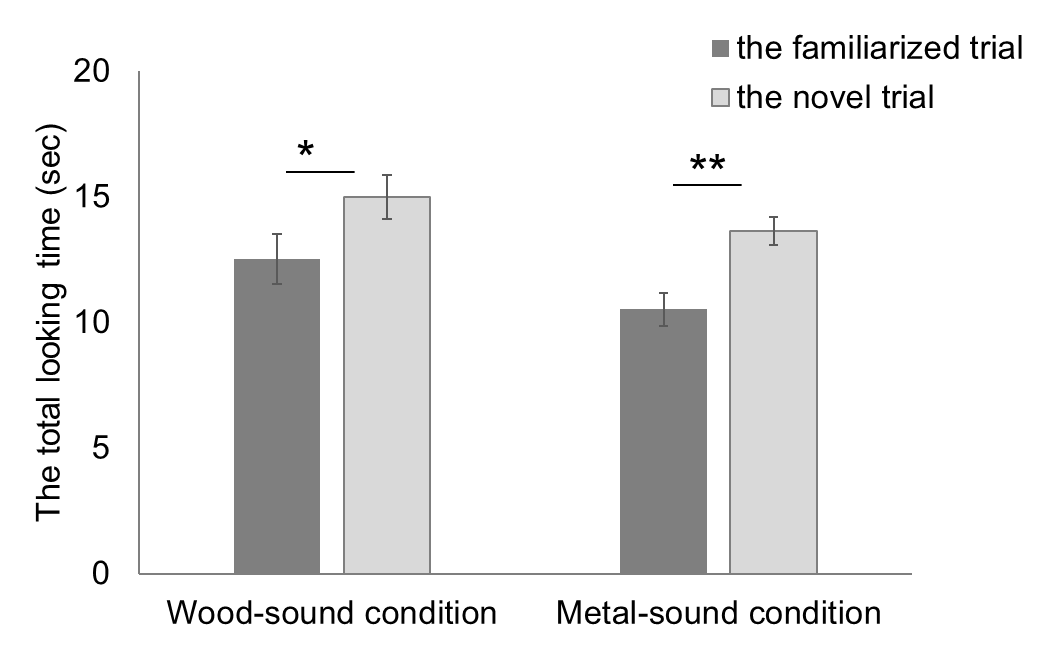


Figure S5. Mean total looking times during the test phase in both the Wood-sound condition and Metal-sound condition. Bars in dark gray and bars in white gray represent the results for the familiarized trial and the novel trails, respectively. The error bars represent ±1 standard error of the mean. Asterisks indicate the significance level of the statistical differences: *P < 0.05 and **P < 0.01.

Supplemental references

S1. Imai, M. *et al*. Sound Symbolism Facilitates Word Learning in 14-Month-Olds. *PLoS ONE* **10**, e0116494 (2015).

S2. Kanero, J., Imai, M., Okuda, J., Okada, H. & Matsuda, T. How sound symbolism is processed in the brain: A study on Japanese mimetic words. *PLoS ONE* **9**, e97905. (2014).
